# The LIM and SH3 domain protein family: structural proteins or signal transducers or both?

**DOI:** 10.1186/1476-4598-7-31

**Published:** 2008-04-17

**Authors:** Thomas GP Grunewald, Elke Butt

**Affiliations:** 1 Department of Pediatrics, Klinikum rechts der Isar, Technische Universität München, Pediatric Oncology Center, Kölner Platz 1, D-80804 Munich, Germany; 2 Institute for Clinical Biochemistry and Pathobiochemistry, University of Wuerzburg, Grombuehlstr. 12, D-97080 Wuerzburg, Germany

## Abstract

LIM and SH3 Protein 1 (LASP-1) was initially identified from a cDNA library of metastatic axillary lymph nodes (MLN) more than a decade ago. It was found to be overexpressed in human breast and ovarian cancer and became the first member of a newly defined LIM-protein subfamily of the nebulin group characterized by the combined presence of LIM and SH3 domains. *LASP2*, a novel *LASP1*-related gene was first identified and characterized *in silico*. Subsequently it proved to be a splice variant of the *Nebulin *gene and therefore was also termed LIM/nebulette. LASP-1 and -2 are highly conserved in their LIM, nebulin-like and SH3 domains but differ significantly at their linker regions. Both proteins are ubiquitously expressed and involved in cytoskeletal architecture, especially in the organization of focal adhesions. Here we present the first systematic review to summarize all relevant data concerning their domain organization, expression profiles, regulating factors and function. We compile evidence that both, LASP-1 and LASP-2, are important during early embryo- and fetogenesis and are highly expressed in the central nervous system of the adult. However, only LASP-1 seems to participate significantly in neuronal differentiation and plays an important functional role in migration and proliferation of certain cancer cells while the role of LASP-2 is more structural. The increased expression of LASP-1 in breast tumours correlates with high rates of nodal-metastasis and refers to a possible relevance as a prognostic marker.

## Domain organization and functional structure of human LASP-1

The *LASP1 *gene was initially identified together with three other genes from a cDNA library of metastatic axillary lymph nodes (MLN) from human breast cancer and therefore called *MLN50*. All four genes were mapped to chromosomal region 17q11-q21.3, a region known to contain the *c-erbB-2 *and the *BRCA1 *oncogene and to be altered in 20–30% of all breast cancers [1,2]. Northern blot analysis revealed that the approximately 4.0 kb long mRNA of *MLN50 *is ubiquitously expressed at basal levels in normal tissue and overexpressed in 8% of all tested human breast cancer tissues (5 of 61). Sequence analysis showed that *MLN50 *encoded a putative protein of 261 residues containing a LIM motif at its amino terminus and a *src *homology 3 (SH3) domain at its C-terminal part. This domain organization defined a new LIM protein subfamily characterized by the combined presence of LIM and SH3 domains [1]. *MLN50 *was termed accordingly: **L**IM **a**nd **S**H3 **P**rotein 1 – in short LASP-1.

The LIM domain is an arrangement of eight cysteine and histidine residues (C-X_2_-C-X_16/23_-H-X_2_-C-X_2_-C-X_2_-C-X_16/21_-C-X_2/3_-C/D/H), is found in a number of vertebrate and invertebrate proteins and known to mediate protein-protein interactions as a modular binding interface [1,3-5]. Although no binding partner for the LIM-domain of LASP-1 has been identified so far, the zinc-finger module in the LIM-domain of LASP-1 is a morphologically and perhaps functionally independent folding-unit of this protein harbouring the possibility of direct binding to DNA [6].

The N-terminal LIM domain is followed by two nebulin-like repeats called R1 and R2 each 35 residues long enabling the protein to bind to F-actin. The actin-binding domains of LASP-1 mediate a direct interaction between LASP-1 and actin at cell membrane extensions [7-12]. The binding of LASP-1 to actin stress fibres is mediated through its interaction with palladin that binds to the SH3 domain of LASP-1. siRNA knock-down of palladin leads to loss of LASP-1 at actin stress fibres and redirection to focal contacts without changing actin filaments. Thus palladin is necessary to recruit LASP-1 to actin stress fibres but not to focal contacts [13].

Via its nebulin-like actin-binding repeats LASP-1 has an additional interaction with kelch related protein 1 (Krp1), a focal adhesion protein involved in pseudopodial elongation and cell migration [14]. The binding between LASP-1 and Krp1 occurs in co-localization to the membrane-bound integrin CD44 and to the adaptor protein Ezrin – both of which mediate the cellular contact to the extracellular matrix and intracellular signal transduction in benign and malign cells [14-16].

The exact cellular function of LASP-1 is not known yet, but the protein has previously been reported to localize within multiple sites of dynamic actin assembly such as focal contacts, focal adhesions, lamellipodia, membrane ruffles, and pseudopodia [1,8,17-19], suggesting that it plays an essential role in actin cytoskeleton organisation at leading edges of migrating cells.

The actin-binding-motifs are followed by a linker-region with several characterized specific phosphorylation residues at serine/threonine and tyrosine that regulate function and localization of the protein. In fact, human LASP-1 is phosphorylated by cAMP- and cGMP-dependent protein kinases (PKA and PKG) at serine 146 [9]. In rabbit parietal cells, elevation of intracellular cAMP by forskolin induced a partial translocation of LASP-1 to the apically directed F-actin rich intracellular canaliculus, which is the site of active HCl secretion [17,18,20]. Lack of gastrin stimulation led to decreased LASP-1 phosphorylation and subsequent lack of HCl secretion without changing total amount of LASP-1 protein [21]. In PTK2 cells, transfected with LASP-1 mutant S146D, the pseudo-phosphorylation resulted in a translocation of the protein from the membrane to the cytosol, followed by reduced cell migration [9].

**Figure 1:**
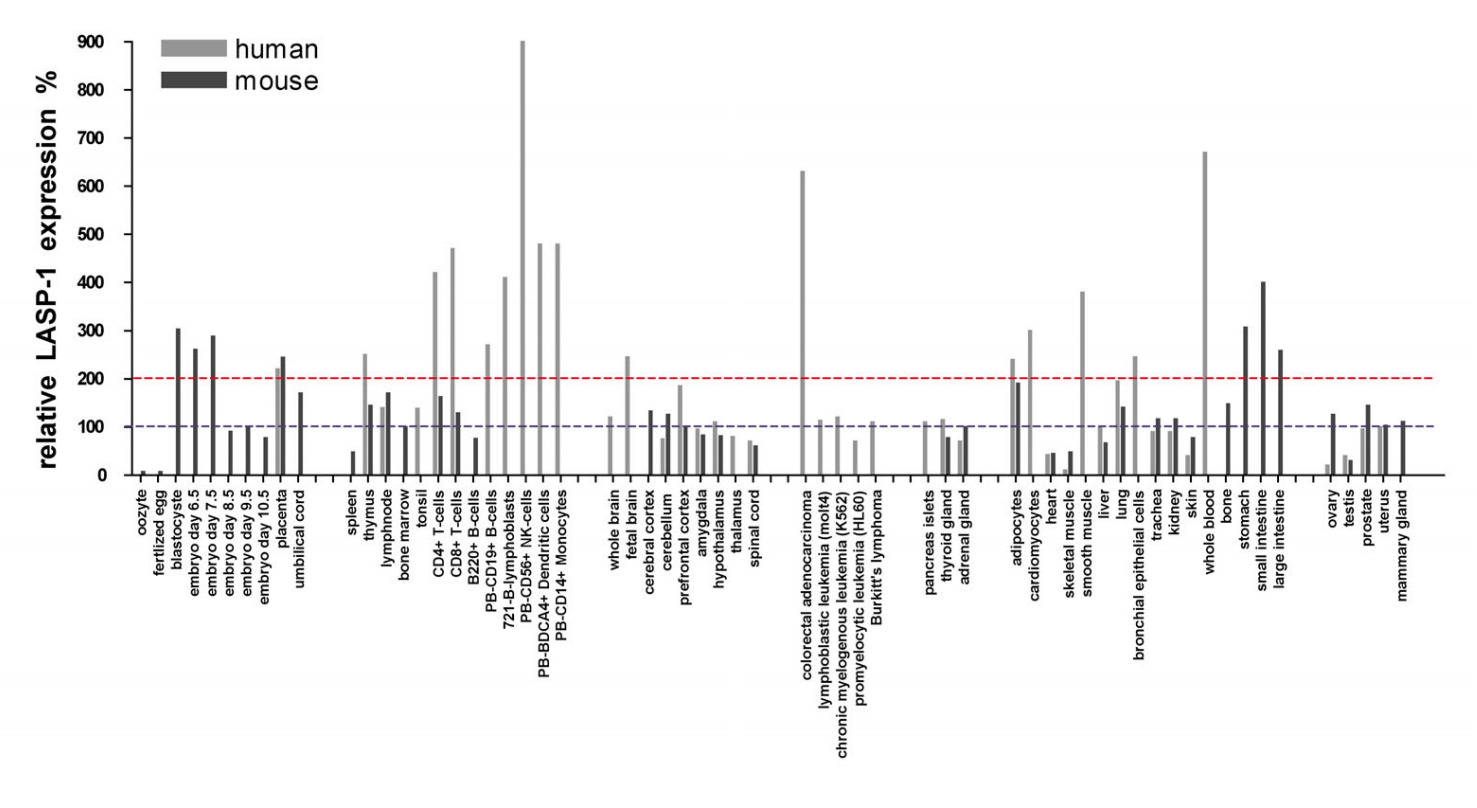
**Relative expression pattern of murine (black columns) and human (gray columns) LASP-1 of selected tissues in part adapted from Su et al. 2002 [22].** The median of LASP-1 expression in all tissue types that have been originally analyzed by Su et al. was set at 100% (blue line); two fold value of the median is indicated in red. As seen in the graph LASP-1 is expressed in a wide variety of human and murine tissues. High to excessive expression is observed during early embryonic development, in immunocompetent cells, fetal brains, muscle cells, entire blood and colorectal carcinomas. Interestingly, LASP-1 is not expressed at high levels in benign tissues of the reproductive tract (ovary and mammary gland), but has been reported to be overexpressed in metastases of malign tumours derived from these tissues.

In contrast to human LASP-1, murine LASP-1 is phosphorylated at threonine 156 by PKA and PKG. Nevertheless, exposure of human and murine mesangial cells to forskolin induced a translocation of both, human and murine LASP-1, from the focal contacts to the cell interior without affecting F-actin structure and a comparison of various murine and human tissues revealed a similar prominent LASP-1 expression (Figure 1) [22]. Altogether, the existing data suggest no functional differences between human and murine LASP-1 [10].

Additionally, human LASP-1 is phosphorylated at tyrosine 171 by Abelson tyrosine kinase [19]. Abelson tyrosine kinase is strongly involved in carcinogenesis of hematopoetic tumours, such as B-cell lymphomas [23]. Phosphorylation at tyrosine 171 is also associated with loss of LASP-1 from focal adhesions and furthermore with the initiation of cell death, but without changes in the dynamics of migratory processes [19].

The C-terminal SH3 motif is a small domain of 60 amino acids, first identified as a conserved sequence in the non-catalytic amino-terminal part of the *src *protein tyrosine kinase. SH3 domain proteins are usually located close to the plasma membrane, suggesting that the SH3 domain may be implicated in localizing the protein to this cell compartment [24].

In fact, LASP-1 primarily localizes to focal contacts, but confocal microscopy and Western blot analysis of cytosolic and nuclear preparations of various breast cancer cell lines also confirmed its nuclear localization [25]. Thus, LASP-1 is not exclusively a cytosolic protein, but is also detectable within the nucleus.

At focal adhesions the C-terminal SH3 domain of LASP-1 is involved in protein-protein interactions through binding to proline-rich sequences, specifically with palladin, lipoma preferred partner (LPP), Prointerleukin-16 (Pro-IL-16), vasodilator stimulated phosphoprotein (VASP) and zyxin [10,26,27,13]. Recent data have shown that after photobleaching of cells and subsequent destruction of cytoskeletal networks, the recovery of LASP-1 and LASP-2 occurred from the anterograde direction while actin recovered inwards from the bundle tips, consistent with the retrograde flow by treadmilling. These results suggest that LASP-1 and -2 participate in the stabilisation of actin-bundles but not in their initiation [11].

Invasion assays with a ΔSH3 deletion mutant of LASP-1 invariably led to the conclusion that especially its SH3 domain is necessary for pseudopodial formation, extension and invasion [14,28].

**Figure 2:**
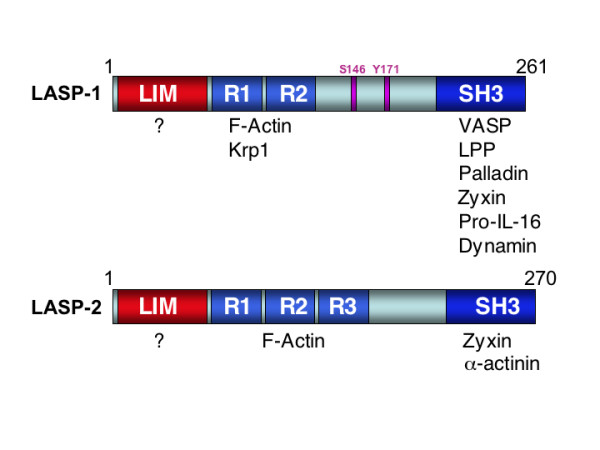
**Schematic models of LASP-1 and LASP-2 domain structures.** Identified binding partners are indicated at the appropriate domains (Krp1 – Kelch related protein 1, LPP – lipoma preferred partner, VASP – vasodilator stimulated phosphoprotein, Pro-IL-16 – Prointerleukin-16). Known phosphorylation sites at serine 146 (S146) and tyrosine 171(Y171) are marked with pink boxes.

In summary, these multilateral protein-protein interactions mediated by the LIM and SH3 domains can be regarded as scaffolds for the formation of protein complexes of higher order and imply that LASP-1 is an important structural protein of the cytoskeleton (Figure 2 and Table 1).

**Table 1:** Summary of putative and potential functions of LASP-1 and LASP-2

**protein**	**localization**	**putative and potential function**	**reference(s)**
**LASP-1**	*focal contacts*	signal transducer for IGF-1	[49]
		zyxin recruiting protein	[38, 39]
		modulator of migration and pseudopodial elongation	[19, 25, 38, 39, 46, 47]
		interaction partner for cytoskeletal organisation	[7, 8, 10, 11, 13, 14, 26, 27]
		stabilisation of actin filament bundles	[11]
		modulator of active HCl secretion in gastric parietal cells	[17, 18, 20, 21, 70]
	*nucleus*	modulator of proliferation in concert with other proteins	[25, 38, 39]
		cell-cycle-control	[26, 38, 39]
		(transcription factor)	[6, 25]
	*postsynaptic/CNS*	axonal growth and differentiation/structural protein/autism linked gene	[40, 42]
	*Z-discs/A-bands*	structural protein (possibly function in myofilament stabilization and assembly)	[12]
**LASP-2**	*focal contacts*	interaction partner for cytoskeletal organisation	[27, 30]
		stabilisation of actin filament bundles	[11]
	*Z-discs*	structural protein/myofilament stabilization and assembly	[12, 37]
	*CNS*	unclear/structural protein	[30]

## Domain organization and functional structure of human LASP-2

A novel *LASP1*-related gene, *LASP2*, was identified and characterized by using bioinformatics. The nucleotide sequence of human *LASP2 *cDNA was determined *in silico *and the protein (270 aa) showed 97.4% and 63.7% total-amino-acid identity with murine LASP-2 and human LASP-1, respectively [29]. Apparent molecular weights of LASP-1 and LASP-2 are 38 kDa and accordingly 34 kDa. *LASP2 *was found to be a splice variant of the *Nebulin *gene and was therefore also termed LIM-nebulette. *LASP2 *has a chimeric gene locus, which might be generated through homologous recombination between the ancestral *LASP2-tem7l-cacnb2 *locus on chromosome 10q12 and the ancestral *nebl-arl8 *locus on chromosome 2q23 displaying a classical example for gene fusion during evolution as one of the mechanisms to generate alternative splice variants [29]. In contrast to sarcomeric nebulette, LASP-2 (LIM-nebulette) is also expressed in non-muscle cells. It displays a modular structure with an N-terminal LIM domain, three nebulin-like repeats and a C-terminal SH3 domain and shows high similarity to LASP-1 (Figure 2) [27,29]. However, the linker domains differ significantly between LASP-2 and LASP-1 while all other domains are highly conserved in chicken, mouse, and human [30].

Like LASP-1, LASP-2 is also predominantly located at focal contacts via its nebulin like repeats mediating binding to F-actin and targeting the protein to focal adhesions [12]. Co-precipitation experiments and a yeast two-hybrid screen revealed the specific interaction with zyxin, in which the SH3 domain of LASP-2 is both necessary and sufficient for zyxin binding [27]. LASP-2 shows a subcellular distribution at focal adhesions similar to LASP-1. Thus, both proteins may play an important role in the organization of focal adhesions in cooperation with zyxin [27] which is a key player for correct assembly and disassembly of focal adhesions [31].

The exact cellular function of LASP-2 has not been fully elucidated yet, but the structural differences of LASP-2 in comparison to LASP-1 imply that its function and binding partners might be distinct from those of LASP-1 (Figure 2 and Table 1).

### LASP-1 and LASP-2 in invertebrates

Genes encoding proteins homologous to *LASP1 *and *LASP2 *were also found in gene-databases of invertebrates such as sea urchin, nematodes and insects. Recently, Terasaki et al. described and characterized LASP in the ascidian *Ciona intestinalis *[32]. Multiple alignments between vertebrate and invertebrate LASP-proteins demonstrated a high homology of their domains and the strong expression of putative ancestral LASP in neural lineage cells and the nerve cord of *Ciona intestinalis *is similar to the high expression of LASP-1 and -2 in the CNS of vertebrates [32]. The phylogenetic analysis suggests that the *LASP1 *and *LASP2 *genes might have been generated during evolution from invertebrates to vertebrates.

### LASP-1 and LASP-2 are ubiquitously expressed and crucial for cellular development and differentiation in vertebrates

Human *LASP1 *and *LASP2 *are located on chromosome 17q12 and 10p12/2q23, respectively [2,29]. The murine gene of *LASP1 *was mapped by in situ hybridization to the 11C-11D region on chromosome 11. The isolation and characterization of murine *LASP1 *cDNA showed that it is highly conserved with its human counterpart. Sequence alignment revealed a high level of identity of *LASP1 *and *LASP2 *genes across species barriers [33].

The murine gene is expressed at basal levels in almost all adult tissues and at high levels during murine embryogenesis from blastocyste stage to day 17.5 as shown by Microarray and Northern blot analysis [22,34], indicating an essential role of *LASP1 *in cellular migration and differentiation (see also Figure 1).

Suitably, *LASP1 *was characterized as one gene out of six inserted into a 185-kb contig sequence flanking a transgene on distal murine chromosome 11 that caused a recessive phenotype with skeletal malformation [35]. A comparison with the corresponding *LASP1 *region on human chromosome 17q12 revealed several small-scale rearrangements during evolutionary divergence within this cluster of densely packed genes, hinting to a crucial function of LASP-1 in embryo- and fetogenesis [35]. In further support of this hypothesis it has been shown that human mesenchymal stem cells start to express LASP-1 in parallel to acquiring the capacity for osteogenic differentiation [36].

### LASP-1 and -2: interaction with the cytoskeleton

LASP-1 and LASP-2 are actin-binding scaffolding proteins [8]. LASP-1 has been reported to regulate cell migration, proliferation and to localize at focal adhesions, along stress fibres and leading edges like lamellipodia, filopodia and pseudopodia [8,9]. LASP-2 co-localizes to actin filament bundles in neuronal cell lines [30] as well as in focal adhesions and at intercalated discs in cardiac myocytes [37].

During cell migration actin is supplied to the extending lamellipodia tips to push the cell forward thereby producing a retrograde flow. This process was revealed by fluorescence recovery after photobleaching (FRAP) with eGFP-actin as well as eGFP-LASP-1 and -2. During the rapid process of exchanging actin bundles LASP-1 and -2 recover from the anterograde direction over actin bundles into the tips of lamellipodia. In addition, a lateral cross-linking of actin filaments by LASP-1 and -2 is visible suggesting a participation in stabilisation of actin bundles [11].

When expressed as a truncated form (LASP-1ΔSH3) LASP-1 remains co-localized with F-actin at the tips of pseudopodia but pseudopodia elongation is suppressed demonstrating the important role of LASP-1 in cell motility [14]. Whether this effect is solely dependent on LASP-1 dysfunction or the disrupted binding of the SH3 domain interacting partners remains to be elucidated.

In non-motile serum-starved cells, LASP-1 is localized to the peripheral edge of the cell. Exposure of the cells to growth factors that activate cell migration caused a rapid (1–2 min) relocalisation of LASP-1 from the periphery to focal adhesions and later on (>15 min) to actin-rich membrane ruffles on the cell surface [11]. Interestingly, depletion of LASP-1 did not cause differences in adhesion and spreading of the cells but significantly reduced the ability of the cells to migrate [19,38,39]. Phosphorylation of LASP-1 at tyrosine 171 by Abl-kinase prevents translocation of LASP-1 to focal contacts [19] while phosphorylation at serine 146 by PKA renders the protein more cytosolic [9].

In striated muscle cells LASP-1 localizes to A-bands and Z-discs implying a role for LASP-1 as a structural protein in contractile cells [12]. Consistently a high expression of LASP-1 was observed in human vascular smooth muscle cells and myoepithelial cells of mammary glands [38,39].

Recently, LASP-2 was detected in heart and skeletal muscle [37]. The protein crosslinks and organizes actin filaments into bundles and directly interacts with the Z-disc protein α-actinin [12,37] suggesting a crucial role in myofilament assembly and stabilisation (Table 1). In contrast to LASP-1, LASP-2 is not detectable at A-bands of striated muscle cells. This disparity in localization might be due to specific structural differences in the linker regions and number of nebulin repeats of these proteins [12] (Figure 2).

### LASP-1 and LASP-2 are highly expressed in the central nervous system

Both, LASP-1 and -2 are highly present in the central nervous system as they are prominently expressed in the fetal and adult brain. Compared to LASP-2, which additionally is highly expressed in striated muscle cells, LASP-1 displays a broader expression pattern also among various non-muscle tissues [30,37,40,41] (Figure 1).

As expected from its structural similarity to LASP-1, LASP-2 possesses actin-binding activity and co-localizes with actin filaments in filopodia of neuroblastoma cells [30].

LASP-1 is expressed in cortex, hippocampus, cerebellum and densely concentrated at the postsynaptic membrane of dendritic spines [40,41]. Although the exact function of LASP-1 in CNS is still unclear a region on chromosome 17 has recently been highlighted in multiple studies as being linked to autism (MIM [209850]). A dense panel of single nucleotide polymorphisms (SNPs) was selected across the linkage peak and analyzed in a trio-based study design. Nominally significant single SNPs and/or haplotype-based association results were detected in 15 genes, of which, *MYO1D*, *ACCN1 *and *LASP1 *stand out as genes with autism risk alleles [42].

Moreover, analysis of the molecular signature in neurons under homocysteic acid induced neuronal stress revealed, that, together with several other proteins well known as risk factors in schizophrenia and neurodegeneration, LASP-1 was modified by phosphorylation. Homocysteic acid induces calcium influx into neurons. On the molecular level this is correlated with fast modification of proteins like phosphorylation and proteolysis. Thus the observed phosphorylation suggests a role of LASP-1 in homocysteic acid induced neurotoxicity [43].

Furthermore, during early differentiation of cultured hippocampal cells LASP-1 initially localizes mainly at the leading sites of growth cones but distributes immediately along the dendritic membranes and subsequently clusters at postsynaptic densities of dendritic spines [40]. Consistently, *LASP1*, but not *LASP2*, belongs to a group of several strongly upregulated genes in response to nerve growth factor (β-NGF) stimulation in phaeochromocytomal PC12 cells, which are commonly used as a cell model for neurite outgrowth [44]. These data indicate that in particular LASP-1 is involved in neuronal differentiation and development (Table 1 and 2).

**Table 2:** Differential expression of LASP-1 in various cell types and known regulators and modifiers of LASP-1-expression

**LASP-1 upregulation**					
					
**species**	**cell type**	**modifier/stimulus**	**fold**	**method**	**ref.**
*bovine*	breast epithelium	lactating mammary tissue	>18	cDNA-Microarray	[45]
*mouse*	prostatic adenocarcinma	Neu (erbB2)-overexpression	3.8	cRNA-Microarray	[71]
	RAW264.7 macrophages	treatment with LPS	3.37	cDNA-Microarray	[72]
	pluripotent mesenchymal C3H/10T1/2 cells	activation of Sonic Hedgehog signalling cascade	1.9	cDNA-Microarray	[53]
	interstitial cells of Cajal	comparison to tunica muscularis	1.89	cDNA-Microarray	[73]
	embryonic fibroblasts	overexpression of IGF-receptor	1.6	Northern Blots	[49]
	FDB1 myeloic leukaemia cells	GM-CSF	1.5	QRT-PCR	[74]
*human*	chronic myeloid leukaemia K562 cells	stimulation of cells with haemin	8	cDNA-Microarray	[75]
	BT-474 breast cancer cells	LASP1 gene amplification (8×)	≈ 8	Northern Blots	[2]
	promyelocytic leukaemia HL60 cells	1'-25'-dihydroxy-cholecalciferole	4.97	cDNA-Microarray	[76]
	podocytes	Wilms tumour suppressor 1 mutation	3.3	2D-DIGE	[28]
	HEK293 cells	overexpression of HNF4α	2.8	cDNA-Microarray	[77]
	30 different commercial and primary breast cancer cell lines	gain on 17q12	>2	cDNA-Microarray	[78]
	E-cadherin mutation	Wnt activity	1.89	cDNA-Microarray	[47]
	MDA-MB435S breast cancer cells	E-cadherin mutation	1.77	cDNA Microarray	[47]
	MCF-7 breast cancer cells	Insulin like growth factor (IGF1)	1.6	cDNA-Microarray	[49]
*porcine*	primary retinal Müller glia cells	in vitro conditions	6.6	2D-Gel/Mass Spectrometry	[79]
*rat*	periaqueductal grey cells	reduced exploratory activity in the elevated plus maze	3.41	cDNA-RDA	[80]
	phaeocytochromal PC12 cells	NGF induced SH2b1β-activity	3.3	cRNA-Microarray	[44]
	alevolar macrophages CRL-2192	pseudohypoxia induced by bismuth	2.1	cDNA-Microarray	[81]
					
**LASP-1 downregulation**					
					
**species**	**cell type**	**stimulus**	**fold**	**technique**	**ref.**

*mouse*	hindbrain	model for smith-lemli-opitz-syndrome	-3.24	cRNA-Microarray	[82]
	mammary epithelium	Prolactin receptor knockout	-1.24	cDNA-Microarray	[83]
	murine embryonic fibroblasts	oncogenic RAS expression in absence of p53	n.d.	cDNA-Microarray	[84]
*human*	chondrocytes	proinflammatory cytokine IL-1β	-2.3	cDNA-Microarray	[85]
	mulitiple myeloma (MM)	transition from MGUS to MM	-1.91	cDNA-Microarray	[86]

### LASP-1 within gastric parietal cell HCl secretion

LASP-1 is widely distributed among various secretory tissues e.g. in epithelial cells of the kidney, in ductal cells of the exocrine pancreas and in gastric parietal cells (Figure 1) [17]. The resting parietal cells contain membranous tubules and vesicles that represent the storage depot of the H^+^/K^+^-ATPase proton pump. Upon stimulation the tubulovesicles fuse with the apical membrane for HCl secretion [20]. In unstimulated gastric parietal cells LASP-1 is localized at the cell cortex (basolateral region). Histamine induced phosphorylation of LASP-1 by PKA induced a partial redistribution of LASP-1 from the cortical membrane to the actin enriched apically-directed intracellular tubuli, the site of active proton transport [8,18]. Movement of the vesicles is in part controlled by dynamin, a large GTPase that regulates the fusion of vesicles with the plasma membrane. In this context, LASP-1 has been identified as a dynamin binding protein implying a regulatory function of LASP-1 in HCl secretion by linking the vesicular trafficking machinery with the cytoskeleton [20] (Table 1).

### LASP-1 is over-expressed in human breast and ovarian cancer

LASP-1 expression has been reported to be increased in metastatic breast and ovarian cancer, suggesting that over-expression of LASP-1 may be involved in the migratory process of cancer cells [1,25]. Knock-down of LASP-1 by RNA-interference in metastatic breast and ovarian cancer cell lines did not lead to induction of apoptosis or necrosis but to strong inhibition of migration and proliferation with cell cycle arrest in G2-phase, while artificial over-expression of LASP-1 in non-neoplastic PTK2 cells resulted in a dramatic acceleration of migration [38,39]. These observations are supported by a case-control study correlating the histological scored expression level of LASP-1, with standard clinicopathological parameters. In this report, LASP-1 expression was significantly higher in invasive human breast carcinomas compared to fibroadenomas with strong cytoplasmatic staining for LASP-1 in 55.4% of the invasive tumours. Although levels of LASP-1 expression did not correlate with histological tumour grading, c-erbB2-, estrogen- or progesterone-expression they did correlate significantly with increased tumour size and rate of nodal-positivity. These data indicate an important role of LASP-1 in proliferation and migration of ovarian and breast cancer cells [25].

In a bovine mammary gland model system, microarray experiments yielded many interesting genes exhibiting differential expression in developing and/or lactating mammary tissue, including oncogenes (*VAV3*, *C-myc*), mediators of apoptosis (Caspase 8), and LASP-1 [45]. Thus we assume that LASP-1 might as well be necessary for proliferation of epithelial cells during physiological human breast development and lactation.

During recent years *LASP1 *was identified in several microarray studies that analysed genes associated with tumour development and cancer progression (overview given in Table 2).

LASP-1 is differentially up regulated in several breast cancer cell lines with E-cadherin-mutations and subsequent accelerated migration. E-cadherin is a cell-cell adhesion molecule and tumour invasion suppressor gene frequently altered in human cancers. Through its cytoplasmic domain it interacts with beta-catenin which in turn interacts with the WNT (wingless) signalling pathway affecting a number of target genes – among them *LASP1 *[46,47]. Anti-WNT1 monoclonal antibodies show promising in vitro effects in cancer treatment [48], maybe mediated through down regulation of LASP-1 and other proteins enhancing anchorage independent migration.

A recent study identified genes associated with insulin-like growth factor-I receptor (IGF-IR)-mediated cellular transformation. In cells overexpressing the IGF-IR, LASP-1 is also overexpressed. MCF-7 breast cancer cells treated with IGF-I exhibit upregulated expression of LASP-1 as well. Expression induction required the activation of the PI3-kinase signalling pathway, suggesting that LASP-1 may mediate IGF-IR function in cancer progression and operates as a signal transducer [49].

In a study, which established a prognostic index for nodal-positive breast cancer, all 20 patients were *LASP1 *positive only differing in mRNA expression levels [50]. However, in patients that died within 5 years after surgery *LASP1 *was found to be one out of five genes with decreased expression levels.

Several additional observations underscore the important role of LASP-1 in cancer: Altered LASP-1 expression was associated with the *MLL *gene in acute myeloid leukemia through forming a new translocation-gene of the *LASP1 *and *MLL *gene in a patient with a high risk of stratification. In this patient two different versions of this new fusion gene t(11;17)(q23;q12-21) were identified: a *5'MLL-3'LASP1 *and the reciprocal *5'LASP1-3'MLL *translocation-gene [51,52].

LASP-1 is also transcriptionally upregulated in response to the morphogen Sonic Hedgehog [53] (Table 2). Disruption of the Hedgehog signalling cascade leads to a number of developmental disorders and plays a key role in the formation of a wide range of human cancers [54].

### LASP-1 binds to LPP and zyxin and is localised in the nucleus

In a recent paper variable expression of LASP-1 in breast cancer was detected. In addition to the reported localization at focal contacts and in the tops of lamellipodia, the authors observed a perinuclear and nuclear distribution of the protein [25]. However, sequence analysis revealed no nuclear localization signal for the classical nuclear import pathway. On the other hand, the zinc-finger containing LIM-domain of LASP-1 offers the possibility of direct binding to DNA [6] and LASP-1 may even form heterodomains to become a nuclear transcription factor [55]. Therefore, LASP-1 binding partners might transport the protein from the cytoplasm to the nucleus.

LASP-1 interacts with LPP, a shuttle protein and transcription factor that transduces signals from focal contacts to the nucleus [10]. LPP is a known interaction partner of the tumour suppressor protein Scrib and both proteins co-localize in cell-cell contacts. This interaction links Scrib to a communication pathway between cell-cell contacts and the nucleus, and implicates LPP in Scrib-associated functions. In various benign and malignant tumours, LPP is present in a mutant form, which permanently resides in the nucleus [56,57]. Although the role of this interaction between LASP-1 and LPP is still unclear, LASP-1 might be embedded into this novel signalling cascade for tumour growth and migration.

In this context it is interesting to note that also the LASP-1 binding partner zyxin has been identified as a differentially transcribed gene in several types of cancer by microarray technology [58].

Zyxin is localized primarily at focal adhesion plaques and is crucial for actin filament polymerization in mammalian cells but also has the ability to shuttle into the nucleus like LPP [31,59].

Silencing of zyxin in HeLa cells resulted in a significant reduction of actin stress fibers [60] whereas under cyclic stretch zyxin only dissociated from focal contacts and accumulated in the nucleus, without affecting vinculin or actin filaments [61]. In genetically zyxin-deficient fibroblasts, the cells display deficits in actin cytoskeleton remodeling [62].

LASP-1 silencing in human breast and ovarian cancer cells led to a diffuse cytoplasmic localization of zyxin without protein loss and without changes in neither vinculin distribution nor actin stress fiber organization, emphasizing the importance of LASP-1 for binding and recruiting zyxin to focal adhesions [38,39].

The loss of zyxin at the sites of focal contacts without changing cellular zyxin protein levels is not restricted to cancer cells but was also observed in human umbilical vein endothelial cells [38]. Interestingly, in these cells zyxin could still be detected along the actin stress fibres, indicating the potential existence of another zyxin-recruiting protein along actin stress fibres [8,9].

In zyxin knock-down experiments neither changes in LASP-1 localization, actin cytoskeleton nor vinculin distribution were detectable indicating that zyxin alone does not change focal adhesion morphology [39]. This is concordant with the fact, that genetically zyxin-deficient fibroblasts even show enhanced adhesion to surfaces and increased integrin expression [62]. In synopsis, LASP-1 and zyxin silencing studies have demonstrated that LASP-1 is necessary and sufficient for recruiting zyxin to focal contacts [38,39] (Table 1).

The decreased cell motility after LASP-1 silencing can be explained by the functional loss of zyxin as a scaffolding protein that facilitates the formation of molecular complexes to promote site-specific actin assembly required for cell migration. This is in agreement with previous findings using a non-genetic approach by injecting a peptide derived from the N-terminus of zyxin to displace zyxin from its normal subcellular location and thus leading to reduced cell migration [63]. On the other hand, the siRNA mediated knock-down of zyxin in SKOV-3 cells had no influence on cell migration [39] while genetically zyxin-deficient fibroblasts displayed enhanced migration [62]. To date these disparate effects have not been fully elucidated but may be due to specific cellular features.

Zyxin also shuttles through the nucleus – most likely by association with other LIM-proteins – and regulates gene transcription [55,59,64]. During mitosis, a fraction of zyxin associates with the tumour suppressor h-warts (LATS1) at the mitotic apparatus [65]. H-warts (LATS1) is a key player in mitosis in mammalian cells and loss of its function disrupts normal cell cycle regulation possibly leading to tumor development [66]. In BT-20, MCF-7 and SKOV-3 cells transfected with LASP-1 specific siRNA, zyxin has been shown to dissociate from focal adhesion plaques and to distribute diffusely into the cytoplasm. It is therefore likely that part of zyxin enters the nucleus, binds to h-warts and leads to G2 cell cycle arrest and inhibition of proliferation as observed after LASP-1 silencing [38,39].

**Figure 3:**
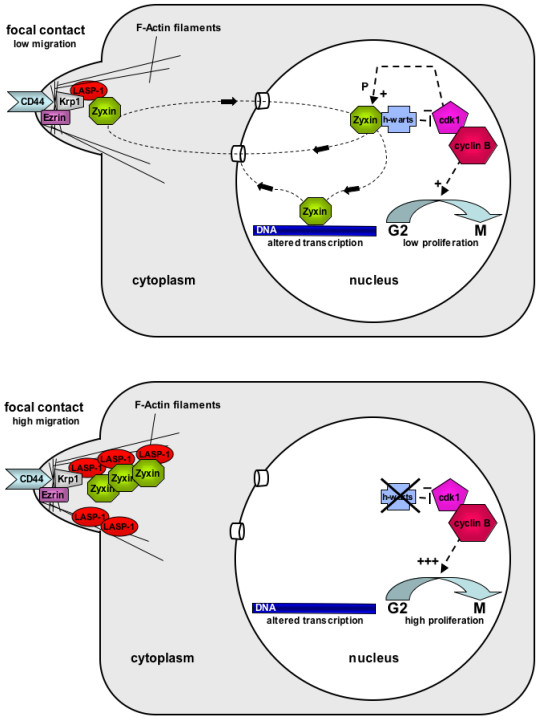
**Model of the putative function of LASP-1 as a zyxin recruiting protein.** Upper panel: physiological situation with normal LASP-1 expression. Lower panel: pathological situation in an LASP-1 overexpressing cell and subsequent disruption of zyxin signalling. CD44 and Ezrin are integrated into this figure as possible upstream interaction partners because of their close colocalization to LASP-1 and Krp1, albeit neither direct nor indirect interaction has been proved yet.

When located at focal contacts, zyxin enhances cell migration [63]. Therefore it is conceivable, that tumour cells overexpressing LASP-1 could recruit more zyxin to focal contacts and thereby contribute to accelerated proliferation and migration of these cells, as higher LASP-1 expression, indeed correlates with metastatic stage and tumour size of human breast cancer [25]. A schematic illustration of this hypothetical functional aspect of LASP-1 is depicted in Figure 3.

Interestingly, in Ewing tumor cells zyxin is only expressed at very low levels and remains diffusely distributed throughout the cytoplasm instead of concentrating in actin-rich dynamic structures. Zyxin gene transfer into EWS-FLI1-transformed fibroblasts leads to inhibition of anchorage independent tumor growth, indicating that zyxin has tumor suppressor activity in these cells [67].

Furthermore, LASP-1 interacts via its SH3-domain with Prointerleukin-16 (Pro-IL-16) [26]. Pro-IL-16 is expressed in both, nucleus and cytoplasm of T cells. Cytoplasmic Pro-IL-16 serves as precursor for mature IL-16 while nuclear pro-IL-16 is associated with G0/G1 cell cycle arrest and with T lymphocyte cell cycle growth suppression [68] indicating a potential role for LASP-1 in modifying T lymphocyte proliferation since it is highly expressed in T-lymphocytes as well [22] (Table 1 and Figure 1)

## Conclusion

To date, an ensemble of more than 50 different structural proteins have been identified that orchestrate the rate and organization of actin polymerization and focal adhesion turnover in protrusion. Moreover, there is a growing number of proteins, that have a dual function in serving as a structural and signalling protein [69]. There are several lines of evidence for LASP-1 being such a dual protein. On the one hand it binds to F-actin and is involved in actin-bundle stabilization [7-9,11], but then shuttles to the nucleus and interacts with LPP [10] and zyxin [27] implying a function in modulating their signalling pathways.

The high degree of structural and amino acid identity between LASP-1 and LASP-2 would suggest that both proteins are functionally redundant, particularly because both proteins are strongly expressed during early embryo- and fetogenesis. However, after expansive study of the literature we conclude that, despite their similarities, LASP-1 and LASP-2 have distinct functions. Although both proteins are expressed in high levels in the CNS, only LASP-1 seems to be significantly involved in neuronal differentiation upon growth factor stimulation and appears to be of importance in epithelial cancer development regarding tumour cell migration and proliferation.

Further studies will be necessary to define the exact physiological functions and to delineate more the differences in function between these highly homologous proteins.

## Competing interests

The authors declare that they have no competing interests.

## Authors' contributions

TG drafted and wrote the paper, designed the tables and the figures. EB corrected and finalized the manuscript. Both authors read and approved the final manuscript
